# A randomised trial assessing the acceptability and effectiveness of providing generic versus tailored feedback about health risks for a high need primary care sample

**DOI:** 10.1186/s12875-015-0309-7

**Published:** 2015-08-05

**Authors:** Natasha Noble, Christine Paul, Mariko Carey, Stephen Blunden, Nicole Turner

**Affiliations:** Priority Research Centre for Health Behaviour and School of Medicine and Public Health, University of Newcastle, Callaghan, NSW 2308 Australia; Casino Aboriginal Medical Service, Casino, NSW 2470 Australia; School of Medicine and Public Health and Department of Rural Health, University of Newcastle, Callaghan, NSW 2308 Australia

**Keywords:** Social disadvantage, Aboriginal Australians, Health risk behaviours, Lifestyle risk factors, Feedback, Tailored, Generic

## Abstract

**Background:**

Tailored feedback has been shown to be effective for modifying health risk behaviours and may aid the provision of preventive care by general practitioners (GPs). However, provision of tailored patient feedback for vulnerable or socially disadvantaged groups is not well explored. The aims of this study were to examine the acceptability and effectiveness of providing generic compared to tailored feedback on self-reported health risk behaviours among a high need sample of people attending an Aboriginal Community Controlled Health Service (ACCHS).

**Methods:**

Participants attending two ACCHSs in regional New South Wales completed a touch screen health risk survey and received either generic or tailored health risk feedback. Participants were asked to complete an exit survey after their appointment. The exit survey asked about feedback acceptability and effectiveness. Self-reported ease of understanding, relevance and whether the generic versus tailored feedback helped patients talk to their GP was compared using Chi-square analysis; The mean number of survey health risks talked about or for which additional actions were undertaken (such as provision of lifestyle advice or referral) was compared using *t-*tests.

**Results:**

Eighty seven participants (36 % consent rate) completed the exit survey. Tailored feedback was rated as more relevant and was more likely to be shown to the participant’s GP than generic feedback. There was no difference in the mean number of health risk topics discussed or number of additional actions taken by the GP by type of feedback.

**Conclusions:**

Tailored and generic feedback showed no difference in effectiveness, and little difference in acceptability, among this socially disadvantaged population. Completing a health risk survey and receiving any type of feedback may have overwhelmed more subtle differences in outcomes between the generic and the tailored feedback. Future work to rigorously evaluate the longer-term effectiveness of the provision of tailored health risk feedback for Aboriginal Australians, as well as other high need groups, is still needed. Trial Registration: Australian New Zealand Clinical Trials Registry ANZCTRN12614001205628. Registered 11 November 2014.

**Electronic supplementary material:**

The online version of this article (doi:10.1186/s12875-015-0309-7) contains supplementary material, which is available to authorized users.

## Background

### High burden of disease associated with modifiable lifestyle risk behaviours

1Chronic diseases including cardiovascular disease, cancer and diabetes, are the leading cause of death and morbidity worldwide [1]. Modifiable risk factors such as smoking, poor nutrition, high blood pressure and cholesterol, physical inactivity and excess alcohol, are key contributors to the development of these chronic diseases [2, 3]. The potential to improve population health and reduce the burden of chronic disease through preventing or reducing lifestyle and biomedical risk factors is therefore significant.

### High risk groups need assistance to address risk factors

For a range of cultural and historical reasons, socially disadvantaged groups, including many indigenous populations, tend to show a higher prevalence of lifestyle risk factors compared to the general population [4, 5]. For example, smoking rates among indigenous populations from Australia, New Zealand, Canada, and the United States (US) far exceed those of their non-indigenous counterparts [6]. In Australia, risk factors including smoking, alcohol misuse, physical inactivity and excess weight also show a distinct association with socioeconomic status, with those in the lowest quintile of socioeconomic disadvantage being almost two times more likely to smoke, and 1.7 times more likely not to exercise, than those in the top quintile [7]. Therefore, there is a need to reduce the prevalence of modifiable risk factors for such populations, if inequities in health outcomes are to be addressed.

### Primary care as a key setting for prevention and management of modifiable risk factors

Primary care is generally the front-line for health care in most countries, and is therefore well placed to address patient risk factors [8]. Each health care visit represents a potential opportunity for the delivery of preventive health care services [9]. However, rates of delivery of preventive care in general practice remain low [10]. Data indicate that risk factors such as excess alcohol, smoking and being overweight are not detected by General Practitioners (GPs) for a significant proportion of general practice patients [8, 11, 12].

Lack of practitioner time is one of the most frequently reported barriers to risk factor detection in the primary care setting [13]. Techniques which enable health care providers to efficiently identify patient risks factors may therefore improve provision of preventive care. Routine screening and delivery of point of care feedback is one technique which may help prompt both health care providers and patients to engage in preventive care. The use of such feedback is well tested in some settings. For example, the provision of tailored health risk feedback to individuals appears to influence change across a range of behaviours including alcohol, smoking and nutrition [14–16]. Similarly, decision support systems which provide recommendations for clinicians have shown benefits for preventive care such as screening, counselling and identification of at risk behaviours [17]. However, the acceptability and effectiveness of providing health risk feedback to socially disadvantaged or vulnerable population groups is not as well explored.

### Tailored versus generic feedback

Feedback can range from simple advice to more intensive or tailored information [14]. Generic feedback contains information which is broadly true for an entire population, such as warnings about alcohol consumption in pregnancy; while tailored feedback is derived from personal information provided by the individual, for example providing information about an individual’s level of drinking compared to a reference group or recommended safe level [14, 18]. Tailored health risk feedback contains less redundant information than generic feedback, and is therefore more likely to be read and remembered by individuals [19]. Evidence also suggests that targeting multiple rather than single health behaviours does not diminish the effectiveness of feedback [20, 21]. The use of pictures that are closely linked to text in health education information has also been found to increase attention and recall, particularly for those with lower literacy levels [22].

### Aims of current study

Aboriginal Community Controlled Health Services (ACCHSs) aim to provide culturally appropriate primary care to Aboriginal people [23], with the majority of those attending being of Aboriginal or Torres Strait Islander origin (74–85 %) [24]. Aboriginal Australians, and potentially also other non-Aboriginal people attending ACCHSs, are an example of a socially disadvantaged group with disproportionately high prevalence of modifiable risk factors [25], for whom the acceptability and effectiveness of provision of health risk feedback has not previously been explored. The aims of the current study were therefore to examine, among patients attending an ACCHS:

- The acceptability of providing generic or tailored feedback on self-reported health risk behaviours, as assessed by i. reported ease of understanding; ii. perceived relevance of the information; and iii. likelihood of the information helping patients to talk to their GP about any relevant survey risk factors; and
- The effectiveness of tailored feedback compared to generic feedback, for prompting discussion or action on relevant risk factors (such as referral or follow up) during the consultation between the GP and patient.

## Methods

### Study design and setting

People attending two ACCHSs in regional New South Wales (NSW, Australia) were invited to complete an anonymous, cross-sectional health risk survey administered on a touch screen computer. Previous work by the authors confirmed that the touch screen survey was highly acceptable to participants in this setting [26]. Appointment sessions (morning or afternoon) were randomised to either the intervention (tailored feedback) or control (generic feedback) condition using a computer algorithm embedded within software developed for the study. The software was run prior to each session to determine session condition. Approximately equal numbers of sessions were randomised to each condition. Randomisation by session was used to minimise contamination between feedback conditions arising from patients potentially sharing their feedback. In general, morning session patients did not overlap with afternoon patients due to a lunch break between sessions. Study recruitment took place over four months in 2012 and 2013. Ethics approval for the study was granted by the University of Newcastle (reference: H-2011–0153) and the Aboriginal Health and Medical Research Council of NSW (reference: 806/11). The CONSORT 2010 checklist of information to include when reporting a randomised trial for this study is included in Additional file 1.

### Participants

Adults (≥18 years) attending the ACCHS for a GP appointment, who were physically and mentally able to provide informed consent and complete the survey, were eligible to participate. Aboriginal and non-Aboriginal people were invited to take part. GPs were informed about the study in person (either via individual [site 1] or group [site 2] meetings) and their consent assumed through agreement of the Chief Executive Officers of the participating services. GPs were shown examples of the two types of feedback but did not receive any specific training regarding responding to the feedback.

### Procedure

**Fig. 1:**
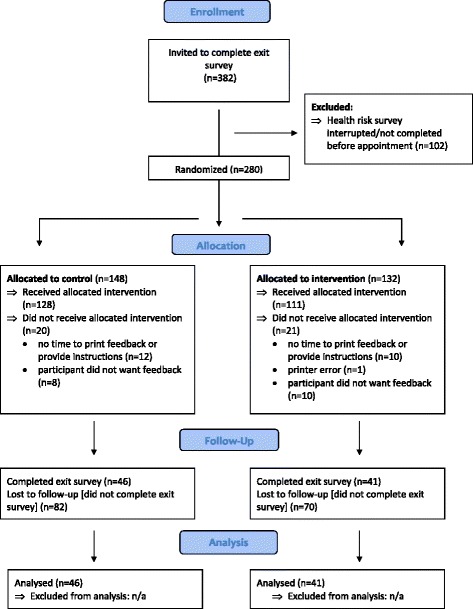
Flow chart of participant recruitment and allocation

Participants were approached by a Research Assistant (RA) in the waiting room and invited to complete a health risk survey while waiting for their appointment. Assistance to complete the survey was offered as required. An Aboriginal RA undertook patient recruitment for half of the recruitment period. Participants were asked to have their weight and height measured (optional), and were able to end the survey if called in for their appointment. After completing the survey, participants were offered printed generic or tailored feedback (depending on session randomisation) and asked to complete a brief exit survey after their GP appointment. An identification slip given to participants was used to link their health risk and exit survey data. Participants were told that they could show the feedback to their doctor if they wanted, and instructed to ask their doctor or health worker if they had any questions about the feedback. It was not possible to blind participants, health care providers or researchers to allocation to intervention condition. A flow chart showing participant recruitment and allocation is shown in Fig. 1.

### Measures

#### Health risk survey

Details about the health risk survey have been published elsewhere [26]. Briefly, the survey assessed self-reported risk factor status for up to 11 key health risks including: body mass index, smoking status, alcohol consumption, physical activity, fruit and vegetable intake, depression, drug use, and time since last screening for blood pressure, cholesterol, diabetes or HbA1c for those with diabetes, and cancers (including cervical, breast and colorectal cancers, according to participant age and gender). A copy of the text of the health risk survey is available in Additional file 2.

#### Exit survey

Participants were asked to complete an eight-item exit survey presented on a second touchscreen computer immediately after their GP appointment. The exit survey asked participants to self-report any of the 11 health risks that they had talked to their GP about during their appointment (including ‘*none of these*’ and ‘*I prefer not to say*’ options). Participants were also asked whether the feedback (called the ‘checklist’) helped them to talk about any of these topics (*Yes- I gave the checklist to the doctor/ Yes- the checklist gave me some ideas about what to ask the doctor/ No- I didn’t use the checklist/ Not sure*), and whether any additional actions were taken by their GP (*Gave me information (e.g., a website or pamphlet)/ Helped me plan changes to my lifestyle/ Organised a follow up appointment for me/* etc.). For the latter two questions a picture illustrating the health risk topics from the survey was shown and participants were asked to answer just for these topics. Finally, participants were asked to evaluate the feedback by indicating whether it: ‘was easy to understand’, ‘was relevant to me’, and ‘will help me improve my health’ (*Yes/ No/ Not sure*). A copy of the text of the exit survey is available in Additional file 3.

### Health risk feedback

Generic feedback included basic lifestyle recommendations and test screening intervals (for those at average risk) based on national guidelines [27–30], for the 11 risk factors covered in the survey. For example, generic feedback recommendations for smoking were: “*If you are a smoker, quitting smoking will improve your health. Talk to your doctor or health worker about ways to quit*”. Tailored feedback was generated by comparing each participant’s survey responses to national guidelines or other accepted cut-offs, and displayed only those factors for which the participant was at risk. Tailored feedback showed the participant’s current status compared to the guidelines (e.g., “*Your weight = 100 kg. A healthy weight for you = 88 kg*”, calculated using a Body Mass Index of 25 kg/m^2^ and participants’ measured height), and listed any screening tests for which the participant was overdue. Both types of feedback included simple advice for improving each risk factor, and used pictures, colour and minimal text to maximise appeal [22]. Feedback design and content was based broadly on public health guidelines (e.g., [30, 31]), as well as input from project collaborators with expertise in Indigenous health and from staff at participating ACCHSs. Examples of the generic and tailored feedback are shown in Additional file 4. At the second site only, a separate ‘GP prompt sheet’ was added to the tailored feedback for patients, based on recommendations from staff at this service. Participants were instructed that they could give this sheet to their doctor if they wanted. The GP prompt sheet consisted of a separate page showing any health topics that a participant indicated they would like more advice about or help with (see also Additional file 4).

### Analysis

Simple proportions and chi-square tests (or Fisher’s exact tests for small cell sizes) were used to assess consent bias and to compare proportions of participants agreeing with statements about the acceptability of the feedback and whether the feedback was useful during their appointment. The mean number of health topics discussed and mean number of actions undertaken for those who received the generic versus the tailored feedback were compared using *t*-tests. Due to the low response rate (see below), results for the tailored feedback at both sites (with and without the additional GP prompt sheet) were combined and analysed as a single intervention condition.

## Results

### Sample

The overall consent rate for the health risk survey was 69 %. There were no significant differences between the age and gender of consenters and non-consenters (p’s > .05; data not shown). Non-Aboriginal people were significantly more likely to consent, compared to the proportions of total active Aboriginal and non-Aboriginal patients registered in the clinical records of the ACCHSs, *χ*2 (1, *N* = 4091) = 9.71, *p* = 0.002. Demographic data confirmed that the total sample represented a broadly socially and economically disadvantaged group, with 66 % reporting Centrelink (government welfare) as their source of income, and the majority of the sample (56 %) having a highest education level of year 10 or below, compared to approximately 35 % of the general Australian population [32] (data not shown).

Of the 239 participants who were given the feedback, 87 completed the exit survey (36 % consent rate). Of these, 46 participants (53 %) received the generic and 41 participants the tailored feedback. The demographic characteristics of those who completed the exit survey are shown in Table 1. There were no significant differences between participants who did and did not complete the exit survey in terms of age, gender or Aboriginal status (all p’s > .05, data not shown).

**Table 1:** Demographic characteristics of participants completing the exit survey by group allocation (*n* = 87)

Demographics	Generic feedback (*n* = 46)	Tailored feedback (*n* = 41)	All exit survey completers
	n (%)	n (%)	n (%)
Gender			
Male	17 (37 %)	18 (44 %)	35 (40 %)
Female	29 (63 %)	23 (56 %)	52 (60 %)
Age			
<35 yrs	15 (33 %)	11 (27 %)	26 (30 %)
35-54 yrs	17 (37 %)	15 (37 %)	32 (37 %)
≥55 yrs	14 (30 %)	15 (37 %)	29 (33 %)
Aboriginal status			
Aboriginal	34 (74 %)	28 (68 %)	62 (71 %)
Non-Aboriginal	12 (26 %)	13 (32 %)	25 (29 %)
Highest level of education^a^			
Yr 10 or below	25 (54 %)	25 (61 %)	50 (58 %)
Yr 12	6 (13 %)	3 (7 %)	9 (10 %)
TAFE/Other	6 (13 %)	6 (15 %)	12 (14 %)
University/Tertiary	9 (20 %)	6 (15 %)	15 (17 %)
Employment status			
Employed	15 (33 %)	17 (42 %)	32 (37 %)
Unemployed/supported	31 (67 %)	24 (59 %)	55 (63 %)

^a^1 missing value

### Patient assessment of the generic and tailored feedback

**Fig. 2:**
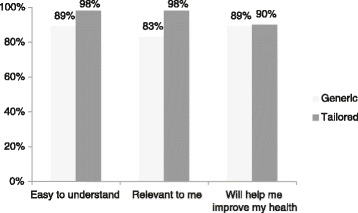
Assessment of the generic and tailored feedback (% who responded ‘yes’ to acceptability statements; *n* = 87)

The percentage of participants agreeing with statements assessing the two types of feedback (i.e., those responding ‘yes’) are shown in Fig. 2. Participants were significantly more likely to agree that the tailored feedback was ‘relevant to me’ compared to the generic feedback, Fisher’s exact: *χ*^2^(1, *N* = 87) = 5.22, *p* = .03, while agreement did not differ for the other statements (*p*s > .05).

### Usefulness of the feedback

For those who reported talking to their GP about any of the health risks in the survey (*n* = 38 generic, *n* = 32 tailored), participants were asked whether the feedback checklist helped them during their appointment. Responses are shown in Table 2. Significantly more participants who received the tailored feedback reported showing this to their GP than those who received the generic feedback, Fisher’s exact *χ*^2^(2, *N* = 70) = 7.30, *p* = .03.

**Table 2:** Proportion of participants using the feedback to talk to their GP about survey health risks

Use of feedback during GP appointment	Generic feedback	Tailored feedback
	n (%)	n (%)
I gave/showed the feedback to the doctor	2 (5 %)	9 (28 %)
The feedback gave me some ideas about what to ask the doctor	14 (37 %)	11 (34 %)
I didn’t use the checklist/not sure	22 (58 %)	12 (38 %)

### Effectiveness of generic versus tailored feedback

The average number of survey health topics talked about during the appointment was 2.85 (SD = 2.33). The majority of participants (82 %) reported talking to their GP about at least one of the survey health risks in their appointment, regardless of whether or not they reported using the feedback. The mean number of topics discussed did not differ between those who received the generic (*M* = 2.87, *SD* = 2.39) versus the tailored feedback (*M* = 2.83, *SD* = 2.28; *t*(85) = 0.08, *p* = .94). There was also no difference in the average number of additional actions related to survey health risks taken by the GP (such as help plan changes to lifestyle, gave me information, organised a follow up appointment) between participants who received the generic versus tailored feedback (*t*(85) = 0.85, *p* = .40).

## Discussion

Tailored feedback was rated as ‘more relevant’, and was more likely to be shown to the GP than the generic feedback. Given that the tailored feedback included personalised risk factor information, it is not surprising that it was rated as more relevant. Almost two-thirds of participants given the tailored feedback indicated that they either showed the feedback to their GP or it gave them some ideas about what to ask the doctor, compared to less than half of those who received generic feedback. Anecdotally, the tailored feedback prompted a greater response from participants than the generic feedback, including several who were surprised by their recommended healthy weight, and one participant who commented that seeing her depression score prompted her to bring this up with her GP. However, participants did not rate the tailored feedback as any more likely to help them improve their health, or any easier to understand, than the generic feedback.

The type of feedback did not appear to influence interaction with the GP in terms of the number of health risks talked about or other actions offered. An average of almost three health survey topics was discussed for all participants completing the exit survey regardless of type of feedback given, and an average of 36 % of these participants indicated that their GP helped them to plan changes to their lifestyle during their appointment (data not shown). It is likely that GPs within the ACCHS setting are already discussing relevant health risks with their patients, as indicated by the majority of participants who reported discussing at least one survey health risk, independently of whether they showed the feedback to their GP. Alternatively, it may be that the process of screening by completing the health risk survey (as suggested by McPhail et al. 2014 [33]), and being provided with feedback, prompted discussion, even if the feedback was not shown to the GP and/or the participant did not report using the feedback.

In contrast to previous findings such as those reported by de Vries et al. [34], and Skinner et al. [16], tailored feedback did not outperform generic feedback in this study. In a meta-analysis of tailored health behaviour change materials, tailored interventions with more than one contact with participants (for example, providing three feedback letters at different times [34]) had significantly larger effect sizes than those with only one point of contact [18]. Also, materials which were tailored to multiple concepts such as participants’ stage of change, and/or self-efficacy, as well as behaviour, were more effective than those tailored on behaviour alone [18]. Thus a more intensive approach to the provision of tailored feedback, such as one tailored to stage of change, or to patient and/or health practitioner perception of risk [35], in addition to behaviour, and using an outcome measure not limited to immediate interaction between the participant and their GP, may have revealed a differential effectiveness of the two types of feedback for people attending an ACCHS. Alternatively, it may be that providing generic feedback to participants provides enough of a trigger to prompt discussion with the GP in this setting.

There were a number of limitations to this study. Firstly, the poor overall response rate and small sample who completed the exit survey substantially limit the power of the study to detect any differences between feedback types. Despite the exit survey being as brief as possible, the majority of participants indicated when invited that they would not have time to complete the exit survey after their appointment. Participants attending the ACCHSs often waited for a long time, came with others, and/or had others waiting for them to finish their appointment. In this setting, an exit survey may not be an effective way to obtain post-appointment data. Alternatively, some kind of incentive may need to be offered to encourage participation. It is likely that those who completed the exit survey had a greater interest in their health, and therefore the results may over-report the usefulness of both the generic and tailored feedback. An objective measure of the interaction between patient and GP (e.g., audio-recording) would also have allowed more systematic identification of relevant health risk discussions (which was limited to any relevant risk a patient reported talking to their doctor about, regardless of the nature or depth of the discussion) or actions. Secondly, there were inconsistencies in providing feedback and inviting participants to complete the exit survey, due to time constraints, participants not wanting feedback, and incomplete surveys (for which tailored feedback could not be generated). These inconsistencies also limit the generalizability of the results as not all eligible participants were invited to complete an exit survey and provide data about the feedback. Lastly, a key limitation involves possible contamination between feedback conditions based on morning or afternoon appointment sessions. As the same practitioners saw patients presenting with both types of feedback, it is possible that advice or action in response to one type of feedback spilled over to patients receiving the other. However, attempting to randomise feedback on another basis would have introduced additional sources of bias such those arising from differences between practitioners or practices.

## Conclusions

Both generic and tailored feedback on multiple risk behaviours appeared to be largely acceptable to this sample of people attending an ACCHS, who are broadly representative of a population experiencing social and economic disadvantage. Tailored feedback was no more effective in prompting health risk discussion or action between participants and their GP than generic feedback. Almost 90 % of participants agreed that either type of feedback would help them improve their health. It is likely that the impact of completing the health risk survey, together with receiving some form of feedback, overwhelmed more subtle differences in outcomes between the generic and the tailored feedback, especially given the small sample size and limited outcome measures. Future work to rigorously evaluate acceptability and longer-term effectiveness of the provision of tailored health risk feedback for Aboriginal Australians, as well as other high need groups, is still needed. Future work may also consider tailoring of feedback to additional concepts such as stage of change or self-efficacy as well as behaviour, and exploring the impact of providing repeated feedback contacts, in assessing feedback effectiveness.

## Additional files

Additional file 1:
**CONSORT 2010 checklist for randomised trials.**
Additional file 2:
**Health risk survey (text version).**
Additional file 3:
**Exit survey (text version).**
Additional file 4:
**Example of the generic and tailored feedback.**

## Abbreviations

ACCHS: Aboriginal Community Controlled Health Service
GP: General Practitioner
NSW: New South Wales
