# Supplementary material for: A randomised trial assessing the acceptability and effectiveness of providing generic versus tailored feedback about health risks for a high need primary care sample
Source: BMC Fam Pract. 2015 Aug 5;16:95. doi: 10.1186/s12875-015-0309-7 (PMC4525725; doi:10.1186/s12875-015-0309-7)
Supplement: Additional file 1: — CONSORT 2010 checklist for randomised trials. [file 12875_2015_309_MOESM1_ESM.doc]

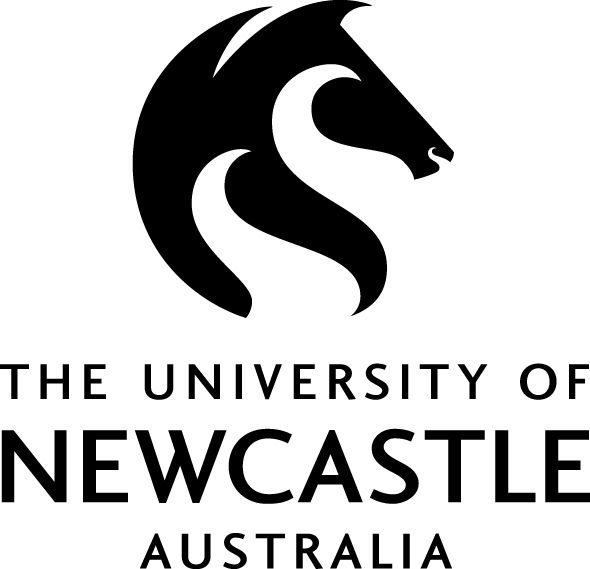


**Healthy Guidelines Checklist**

**TALK TO YOUR DOCTOR OR HEALTH WORKER BEFORE YOU MAKE ANY BIG CHANGES TO YOUR LIFESTYLE**

**Some of these may apply to you:**

| Smoking  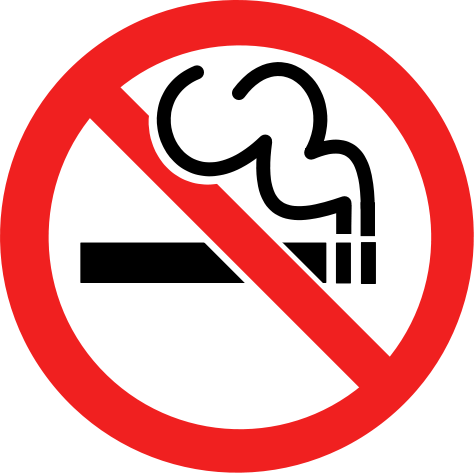 | If you are a smoker, quitting smoking will improve your health.  Talk to your doctor or health worker about ways to quit. |
| --- | --- |
| Fruit and Vegetables  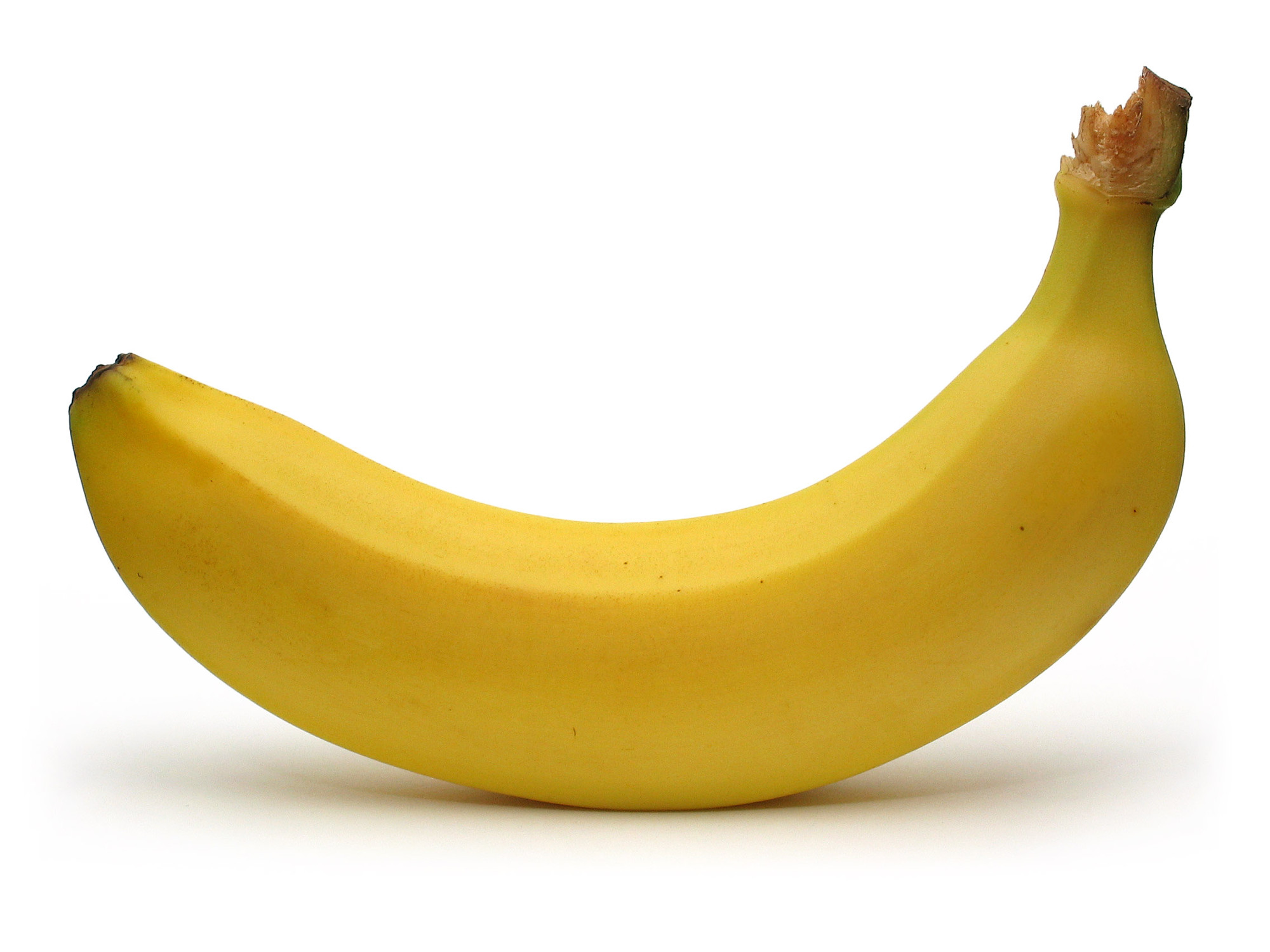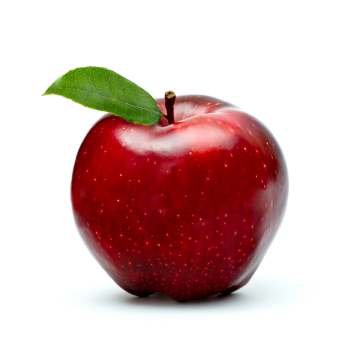  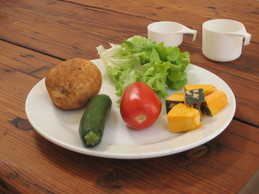 | National guidelines recommend that healthy adults eat:  2 serves of fruit each day  5 serves of vegetables each day  (2 ½ cups of cooked vegies or 5 cups of salad) |
| Exercise  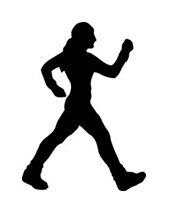 | National guidelines recommend that healthy adults:  Do at least 30 minutes of moderate exercise most days. |
| Alcohol  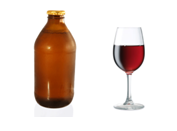 | National guidelines recommend:  Only two standard drinks per day to avoid diseases like cancer.  Talk to your doctor or health worker about ways to limit alcohol. |
| Depression  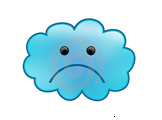 | If you feel sad or depressed a lot, talk to your doctor or health worker. |
| Drug Use  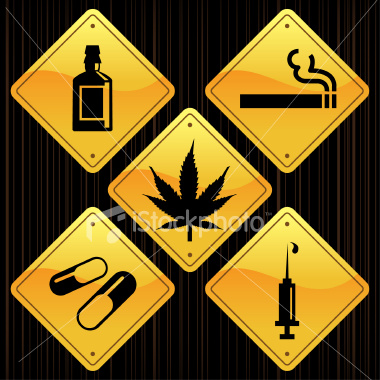 | If you are concerned about your drug use, talk to your doctor or health worker. |
| Weight  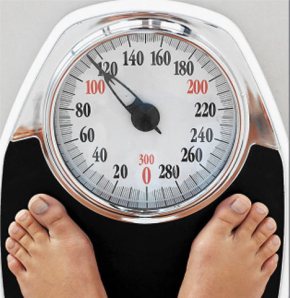 | Keeping a healthy weight is important.  Ask your doctor or health worker whether you need to lose weight. |

**Your GP or Health Worker may be able to give you advice or tell you about other people or programs to help you improve your health.**

**SOME SCREENING TESTS RECOMMENDED FOR PEOPLE AT AVERAGE RISK:**

| **Which test?** | **Who should have the test?** | **How often?** |
| --- | --- | --- |
| Breast cancer check (Mammogram) | All women aged from 50-69 yrs | Every 2 years |
| Cervical cancer check  (Pap smear test) | All women aged 18 yrs and older | Every 2 years |
| Bowel cancer check  (Faecal Occult Blood Test) | Anyone aged 50-75 yrs | Every 2 years |
| Blood pressure check | Indigenous people with diabetes, high blood pressure or kidney disease  Indigenous people, and non-Indigenous people aged 50 yrs or older  Non-Indigenous people | Every 6 months  Every year  Every 2 years |
| Blood cholesterol test | All Indigenous people  Non-Indigenous people aged 45 yrs and older | Every year  Every 5 years |
| Diabetes check  (Fasting blood sugar test) | All Indigenous people aged 35 yrs and older | Every 2 years |
| Blood sugar test  (HbA1c test) | All people with diabetes | Every 6 months |

**Some people who have more risks might need to have the tests more often.**

**PLEASE TALK TO YOUR DOCTOR OR HEALTH WORKER ABOUT WHAT TESTS YOU MIGHT NEED.**


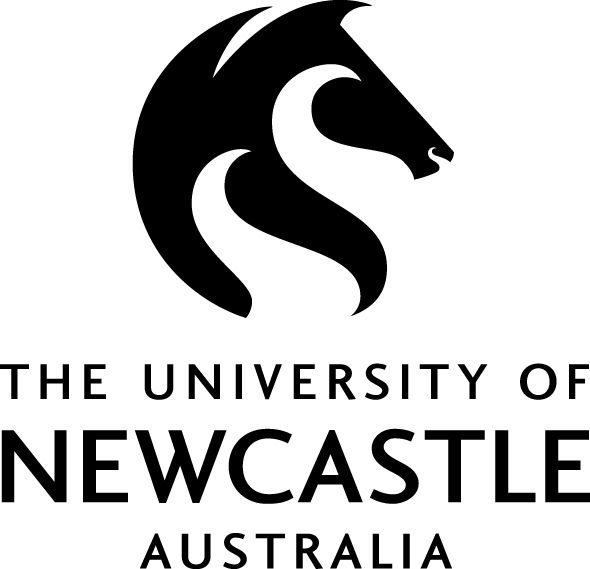


**FOR YOU:**

**Your Health Checklist**

**BASED ON YOUR SURVEY ANSWERS, THESE THINGS COULD BE AFFECTING YOUR HEALTH.**

You might want to talk to your doctor or health worker about:

| **Health Issue** | **What Can I Do?** |
| --- | --- |
| Smoking  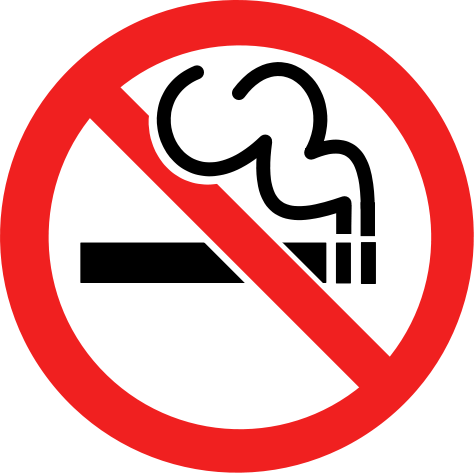 | - Stopping smoking for good will help your health. - If you want to quit, talk to your doctor. - Medicines can help you stop. Ask your doctor, health worker or pharmacist.   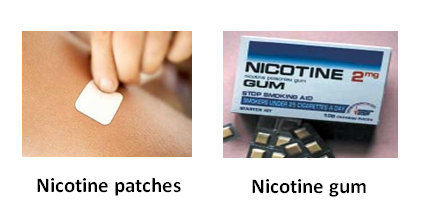   - Your smoking can affect others. Avoid smoking indoors, in the car or near children.   🗸 Feel fitter and have more energy for your family  🗸Save money on cigarettes $$$ |
| Eating enough Fruit and Vegetables  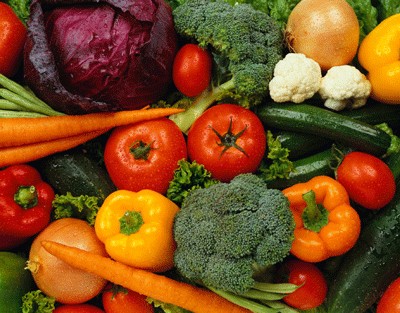  **You have X serves of fruit & X of vegetables a day** | - Eat 2 or more pieces of fruit per day. - Eat at least 5 serves of vegetables a day.   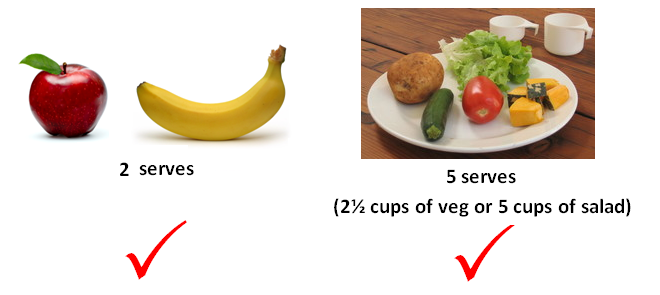   - Have some fruit like an apple or orange as a snack. - Add some vegetables or a salad to your meal.   🗸Help prevent heart disease, stroke and some cancers |

| Not enough exercise  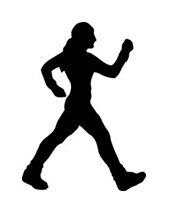 | - Do 30 minutes of exercise every day. - This can be 3 x 10min sessions.   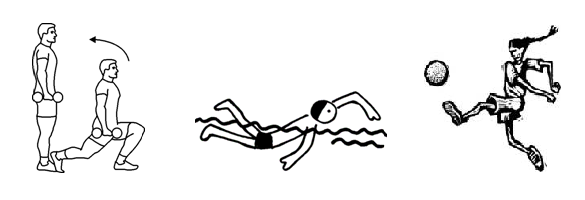   - Try going for a walk with friends or the kids or walking to the shop instead of driving.   🗸Have more energy for your family  🗸Help to lose weight |
| --- | --- |
| Your alcohol use  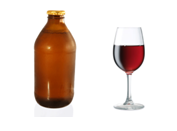  **You <sometimes drink more than 4 drinks on one occasion> or <often drink more than 2 drinks every day>** | - Have no more than 4 drinks in one session. - Try to have 2 or less drinks a day.   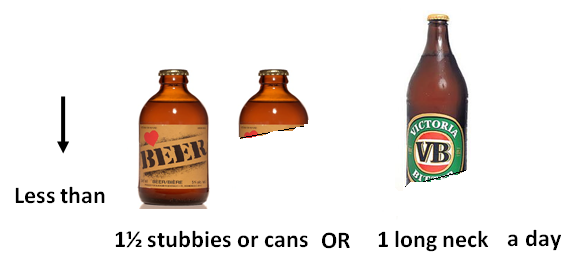   - Try drinks with less alcohol, like light beer. Drink water in between alcohol drinks.   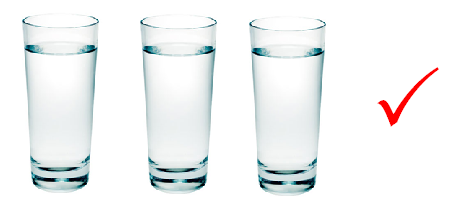  🗸 Better for your brain and your liver  🗸 Less risk of hurting yourself or others |
| Depression  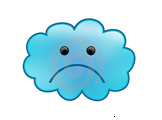  **Your score =** **X**  You might have <mild/mod/severe> depression | - 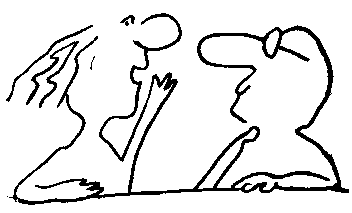If you feel sad, depressed or hopeless a lot, talk to your doctor, health worker or a friend. - Ask to see a counsellor. - Call Lifeline on 13 11 14. - Beyond Blue can also help. Call 1300 224636. |

| Your drug use  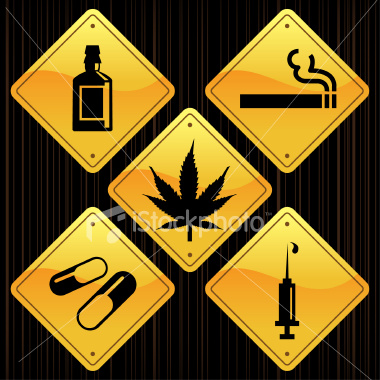 | - Get help to avoid using drugs. - Ask to see the Drug and Alcohol Health Worker. - 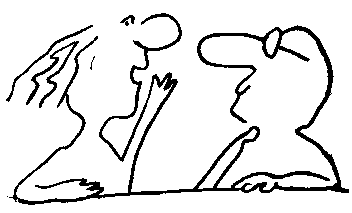Try other support services:   🗸Counselling Online (anytime 7 days per week): [www.counsellingonline.org.au/en/](http://www.counsellingonline.org.au/en/)  🗸Cannabis information & helpline (11am-8pm Mon-Fri): Call 1800 30 40 50. |
| --- | --- |
| Your weight  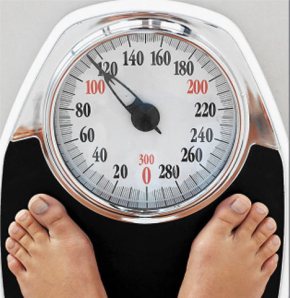  **Your weight = X**  **A healthy weight for you = XX** | - Eat plenty of fruit and vegetables. Eat less fatty, fried or sugary foods.   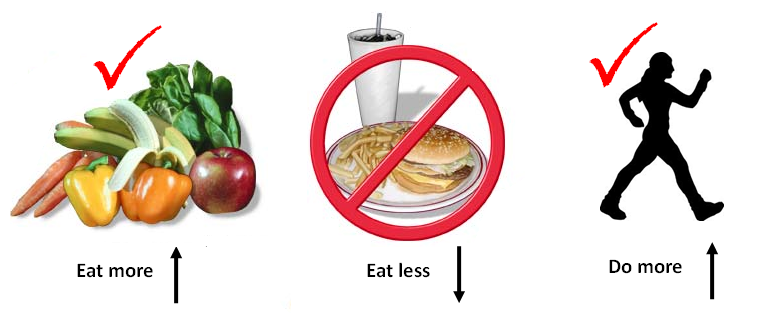   - Do more exercise like going for a walk, or doing a sport. - Call the ‘Get Healthy Information and Coaching Service’ (Mon- Fri 8am – 8pm) on 1300 806 258.   🗸Be healthier for your family  🗸Less risk of getting diabetes |

**Your doctor or health worker may be able to give you advice or tell you about other people or programs to help you improve your health.**

**TALK TO YOUR DOCTOR OR HEALTH WORKER BEFORE MAKING ANY BIG CHANGES.**

**SOME TESTS YOU MIGHT NEED:**

***Ask your doctor of health worker if you need any of these tests***

1. Breast cancer test (a mammogram)

2. Cervical cancer test (a pap smear test)

3. Bowel cancer test (a Faecal Occult Blood Test)

4. Blood pressure check

5. Blood cholesterol test

6. Blood test for diabetes (a fasting blood sugar test)

7. Blood sugar test (HbA1c test to measure your blood sugars over the last month

| ***THIS SECTION IS FOR YOUR DOCTOR.***  ***[Your doctor can give you advice or help you start to make these health changes]*** | | | |
| --- | --- | --- | --- |
| **I WOULD LIKE ADVICE OR HELP FOR:** | **ADVICE ONLY** | **HELP IN THE NEXT MONTH** | **HELP IN A FEW MONTHS** |
| Losing weight | 🗆 | 🗆 | 🗆 |
| Quitting or cutting down smoking | 🗆 | 🗆 | 🗆 |
| Drinking less alcohol | 🗆 | 🗆 | 🗆 |
| Getting more exercise | 🗆 | 🗆 | 🗆 |
| Eating more fruit and veg | 🗆 | 🗆 | 🗆 |
| Stopping or cutting down on drug use | 🗆 | 🗆 | 🗆 |
| Depression or feelings of sadness | 🗆 | 🗆 | 🗆 |
