# Supplementary material for: A randomised trial assessing the acceptability and effectiveness of providing generic versus tailored feedback about health risks for a high need primary care sample
Source: BMC Fam Pract. 2015 Aug 5;16:95. doi: 10.1186/s12875-015-0309-7 (PMC4525725; doi:10.1186/s12875-015-0309-7)
Supplement: Additional file 3: — Exit survey (text version). [file 12875_2015_309_MOESM3_ESM.rtf]

English text of the survey: Durri ACMS Health Risk Survey 2013(1  Question Name = Day of the week)Day of the week:      Monday      Tuesday      Wednesday      Thursday      Friday(2  Question Name = Participant ID)Insert Participant ID:(3  Question Name = Info Screen)(4  Question Name = Gender)Please touch your answer, then touch NEXTSection 1: About You and Your HealthAre you:      Male      Female(5  Question Name = Age)Please touch your answer, then touch NEXTWhat is your age?      18- 24 years      25- 29 years      30- 34 years      35- 39 years      40- 44 years      45- 49 years      50- 54 years      55- 59 years      60- 64 years      65- 69 years      More than 70 years(6  Question Name = Weight kgs)Please touch the numbers on the number pad, then touch NEXT.What is your weight (kg)?(7  Question Name = Height cm)Please touch the numbers on the number pad, then touch NEXTWhat is your height (cm)?(8  Question Name = Indigenous status)Please touch your answer, then touch NEXTAre you of Aboriginal or Torres Strait Islander origin?      Yes, Aboriginal      Yes, Torres Strait Islander      Yes, both Aboriginal and Torres Strait Islander      No(9  Question Name = Diagnosed Conditions)Please touch the boxes for each condition you have, then touch NEXTHave you ever been told by a doctor or health worker that you have any of these conditions?(10  Question Name = Diabetes type)Please touch your answer, then touch NEXTWhat type of diabetes were you told you have?      TYPE 1                                           (usually starts in childhood, needs daily insulin injections)      TYPE 2                                 (usually starts in adulthood, may not need insulin injections)      GESTATIONAL diabetes                     (occurs during pregnancy)      Not sure(11  Question Name = Cancer type Men)Please touch your answer, then touch NEXTWhat type of cancer were you told you have?      Colorectal or bowel cancer      Prostate cancer      Other type of cancer(12  Question Name = Cancer type Women)Please touch your answer, then touch NEXTWhat type of cancer were you told you have?      Breast cancer      Cervical cancer      Colorectal or bowel cancer      Other type of cancer(13  Question Name = Self Report BMI(1))Please touch your answer, then touch NEXTHow would you describe your weight?      Underweight      Normal weight      Overweight      Very overweight or Obese(14  Question Name = Smoking)Please touch your answer, then touch NEXTWhich of the following best describes your smoking?      I smoke daily      I smoke occasionally (e.g. when I have a drink)      I don't smoke now but I used to      I have tried it, but have never smoked more than a total of 100 cigarettes      I have never smoked(15  Question Name = Exercise)Please touch your answer, then touch NEXTDo you usually do at least half an hour of moderate or vigorous exercise on most days (5 or more days) of the week?      Yes      No      Sometimes      Not sure(16  Question Name = Fruit)Please touch your answer, then touch NEXTHow many serves of fruit do you usually eat each day?      None      1      2 or more(17  Question Name = Veg)Please touch your answer, then touch NEXTHow many serves of vegetables do you usually eat each day?      None      1      2      3      4      5 or more(18  Question Name = Alcohol)Please touch your answer, then touch NEXTDo you drink alcohol?      Yes      No      I don't drink now but I used to(19  Question Name = Alcohol 4SDs)Please touch your answer, then touch NEXTHow often do you have MORE THAN 4 standard drinks on one occasion?      Never      Less than monthly      Monthly      Weekly      Daily or almost daily(20  Question Name = Alcohol 2SDs)Please touch your answer, then touch NEXTHow often do you have MORE THAN 2 standard drinks in one day?      Never      Less than monthly      Monthly      Weekly      Daily or almost daily(21  Question Name = Alcohol in past week)Please touch your answer, then touch NEXTIn the last WEEK, did you have any drinks containing alcohol?      Yes      No(22  Question Name = Alcohol Diary Yest)Please try to estimate how many standard drinks you had using the pictures.These questions are about your drinking in the last week. How many standard drinks did you have YESTERDAY?      none      1      2      3      4      5      6      7      8      9      10 or more(23  Question Name = Alcohol Diary THURS)Please try to estimate how many standard drinks you had using the pictures.How many standard drinks did you have on THURSDAY?      none      1      2      3      4      5      6      7      8      9      10 or more(24  Question Name = Alcohol Diary WED)Please try to estimate how many standard drinks you had using the pictures.How many standard drinks did you have on WEDNESDAY?      none      1      2      3      4      5      6      7      8      9      10 or more(25  Question Name = Alcohol Diary TUES)Please try to estimate how many standard drinks you had using the pictures.How many standard drinks did you have on TUESDAY?      none      1      2      3      4      5      6      7      8      9      10 or more(26  Question Name = Alcohol Diary MON)Please try to estimate how many standard drinks you had using the pictures.How many standard drinks did you have on MONDAY?      none      1      2      3      4      5      6      7      8      9      10 or more(27  Question Name = Alcohol Diary SUN)Please try to estimate how many standard drinks you had using the pictures.How many standard drinks did you have on SUNDAY?      none      1      2      3      4      5      6      7      8      9      10 or more(28  Question Name = Alcohol Diary SAT)Please try to estimate how many standard drinks you had using the pictures.How many standard drinks did you have on SATURDAY?      none      1      2      3      4      5      6      7      8      9      10 or more(29  Question Name = Alcohol Diary FRI)Please try to estimate how many standard drinks you had using the pictures.How many standard drinks did you have on FRIDAY?      none      1      2      3      4      5      6      7      8      9      10 or more(30  Question Name = Alcohol Diary THUR1)Please try to estimate how many standard drinks you had using the pictures.How many standard drinks did you have on THURSDAY?      none      1      2      3      4      5      6      7      8      9      10 or more(31  Question Name = Alcohol Diary WED1)Please try to estimate how many standard drinks you had using the pictures.How many standard drinks did you have on WEDNESDAY?      none      1      2      3      4      5      6      7      8      9      10 or more(32  Question Name = Alcohol Diary TUES1)Please try to estimate how many standard drinks you had using the pictures.How many standard drinks did you have on TUESDAY?      none      1      2      3      4      5      6      7      8      9      10 or more(33  Question Name = Alcohol Diary MON1)Please try to estimate how many standard drinks you had using the pictures.How many standard drinks did you have on MONDAY?      none      1      2      3      4      5      6      7      8      9      10 or more(34  Question Name = Diary same as usual)Please touch your answer, then touch NEXTIs this about the same amount of alcohol you would usually drink in a week?      Yes      No, this is MORE than I usually drink      No, this is LESS than I usually drink(35  Question Name = Illicit drugs)Please remember your answers are anonymous. You can also skip this question.When was the last time you used any illegal or illicit drugs?      In the last month (the last 4 weeks)      More than 1 month ago but in the last year      More than 1 year ago (including many years ago)      Never      I prefer not to answer(36  Question Name = Illicit drugs2)Your answers are anonymous. You can skip this question if you want.How often do you usually use illicit or illegal drugs?      Every day      5-6 days a week      3-4 days week      1-2 days a week      Several times a month      About once a month      Less than once a month(37  Question Name = Depression PHQ2)These next questions are about your feelings and emotions.Over the last 2 weeks, how often have you been bothered by the following problems?      Not enjoying things like you used to      Feeling down, sad, depressed or hopeless      never      a little      a lot      all the time(38  Question Name = Depression PHQ9a)Over the past 2 weeks, how often have you been bothered by any of the following problems?      Trouble falling asleep or staying awake, or sleeping too much      Feeling tired or having little energy      Eating more or less than you used to      Feeling bad about yourself. Feeling shamed or that you have let yourself or others down      never      a little      a lot      all the time(39  Question Name = Depression PHQ9b)Over the past 2 weeks, how often have you been bothered by any of the following problems?      Trouble paying attention to what is going on around you      Moving or speaking so slowly that others could have noticed. Or the opposite- being so nervous or restless that you have been moving around a lot more than usual      Thoughts that you would be better off dead or of harming yourself in some way      Feeling angry      never      a little      a lot      all the time(40  Question Name = Blood pressure 6m Indig)Please touch your answer, then touch NEXTWhen did you last have your blood pressure checked?      Never      In the last 6 months (since Dec 2012)      More than 6 months ago (before Dec 2012)      Can't remember(41  Question Name = Blood pressure 6m non-Indig)Please touch your answer, then touch NEXTWhen did you last have your blood pressure checked?      Never      Within the last 6 months (since Dec 2012)      More than 6 months ago (before Dec 2012)      Can't remember(42  Question Name = Blood pressure 12 months)Please touch your answer, then touch NEXTWhen did you last have your blood pressure checked?      Never      In the last year (since June 2012)      More than 1 year ago (before June 2012)      Can't remember(43  Question Name = Blood pressure 2 yrs)Please touch your answer, then touch NEXTWhen did you last have your blood pressure checked?      Never      In the last 2 years (since June 2011)      More than 2 years ago (before June 2011)      Can't remember(44  Question Name = Blood cholesterol 12m Indig)Please touch your answer, then touch NEXTWhen did you last have your blood cholesterol checked?      Never      In the last year (since June 2012)      More than 1 year ago (before June 2012)      Can't remember(45  Question Name = Blood cholesterol 12m non-Indig)Please touch your answer, then touch NEXTWhen did you last have your blood cholesterol checked?      Never      In the last year (since June 2012)      More than 1 year ago (before June 2012)      Can't remember(46  Question Name = Blood cholesterol 2 yr)Please touch your answer, then touch NEXTWhen did you last have your blood cholesterol checked?      Never      In the last 2 years (since June 2011)      More than 2 years ago (before August 2010)      Can't remember(47  Question Name = Blood cholesterol 5 yrs)Please touch your answer, then touch NEXTWhen did you last have your blood cholesterol checked?      Never      In the last 5 years (since mid 2008)      More than 5 years ago (before mid 2008)      Can't remember(48  Question Name = Blood sugar 2 yrs)Please touch your answer, then touch NEXTWhen did you last have your blood sugar level checked?      Never      In the last 2 years (since June 2011)      More than 2 years ago (before June 2011)      Can't remember(49  Question Name = Blood sugar 3 yrs)Please touch your answer, then touch NEXTWhen did you last have your blood sugar level checked?      Never      In the last 3 years (since June 2010)      More than 3 years ago (before June 2010)      Can't remember(50  Question Name = HbA1c test)Please touch your answer, then touch NEXTWhen did you last have a blood sugar (an HbA1c) test?      Never      In the last 6 months (since Dec 2012)      More than 6  months ago (before Dec 2012)      Can't remember(51  Question Name = Mammogram)Please touch your answer, then touch NEXTWhen did you last have a test for breast cancer (a mammogram)?      Never      In the last 2 years (since June 2011)      More than 2 years ago (before August 2010)      Can't remember(52  Question Name = Hysterectomy)Please touch your answer, then touch NEXTHave you had a total or partial hysterectomy?      Yes      No(53  Question Name = Pregnancy)Are you pregnant?      Yes      No      Not sure(54  Question Name = Pap test)Please touch your answer, then touch NEXTWhen did you last have a test for cervical cancer (a Pap smear test)?      Never      In the last 2 years (since June 2011)      More than 2 years ago (before June 2011)      Can't remember(55  Question Name = FOBT)Please touch your answer, then touch NEXTWhen did you last have a test for bowel cancer (a Faecal Occult Blood Test)?      Never      In the last 2 years (since June 2011)      More than 2 years ago (before June 2011)      Can't remember(56  Question Name = Colonoscopy)Please touch your answer, then touch NEXTHave you had a colonoscopy in the last 5 years (since mid 2008)?      Yes      No      Can't remember(57  Question Name = Colonoscopy symptom)Please touch your answer, then touch NEXTDid you have your last colonoscopy because you had a symptom of bowel cancer?      Yes      No(58  Question Name = Health Assess)When did you last have a Health Assessment?      Never      In the last year (since June 2012)      More than 1 year ago (before June 2012)      Can't remember(59  Question Name = Previous advice1)Please touch all that applyHas a doctor ever talked to you or given you advice about any of these things?      weight      smoking      exercise      diet      alcohol      drugs      depression      None of these(60  Question Name = Previous advice2)Please touch all that applyHas a doctor ever talked to you or given you advice about any of these things?      Blood pressure      Blood cholesterol      Diabetes      Tests for cancer      None of these(61  Question Name = Marital status)Section 2: About YouWhat is your marital status?      Single      Married or living with a partner (defacto)      Divorced or separated      Widowed      Other(62  Question Name = Education)What is the highest level of education you have completed?      Primary school      Some high school (year 9 or below)      Year 10 (at school or TAFE)      Year 12 (at school or TAFE)      TAFE course      University or other tertiary qualification      Other(63  Question Name = Income source)What is your main source of income?      Centrelink (e.g. Newstart, Disability pension or Abstudy)      Part-time or casual employment      Full time employment      Supported by a partner or other family member      Other(64  Question Name = Payday)Was payday in the last 7 days?      Yes      No      Not sure(65  Question Name = Practice visits)About how many times in the past 12 months have you come to this practice to see a doctor or Health Worker (not including this visit today)?      None      1      2      3      4      5      6      7      8      9      10 or more(66  Question Name = Housing)Which best describes the type of housing where you live?      Privately rented      Rented from the government (Housing Commission)      Owned or being paid off      Boarding      Housing provided by work      Staying with family      Other(67  Question Name = No. Adults)How many OTHER adults (18yrs +) usually live in the same house as you?      None      1      2      3      4      5 or more(68  Question Name = No. Children)How many children (under 18 yrs) usually live in the same house as you?      None      1      2      3      4      5 or more(69  Question Name = No. Bedrooms)How many bedrooms are there in your house?      None      1      2      3      4      5 or more(70  Question Name = Physical force)In the last 12 months, did anyone, including people you know, use physical force or violence against you?      Yes      No(71  Question Name = Emotional)In the last 12 months, did anyone, including people you know, use emotional violence against you e.g. insult you, swear or scream at you, or threaten to hurt you?      Never      Sometimes      Often(72  Question Name = Want GP advice)Please touch any topics you would like advice aboutSECTION 3: HEALTH CHANGESWould you like advice from your doctor about:      weight      smoking      exercise      diet      alcohol      drugs      depression      None of these(73  Question Name = Want GP advice2)Please touch any topics you would like advice aboutWould you like advice from your doctor about:      Blood pressure      Blood cholesterol      Diabetes      Tests for cancer      None of these(74  Question Name = Hardest change)Please choose ONE optionWhich of these health changes would be the HARDEST for you to make?      Lose weight      Stop or cut down smoking      Drink less alcohol      Get more exercise      Eat more fruit and veg      Stop or cut down on drug use      Find ways to feel less sad or depressed      Not sure(75  Question Name = Changes want to make)Please choose as many changes as you likeIf you could get help, are there any of these changes you would like to make?      lose weight      stop or cut down smoking      drink less alcohol      get more exercise      eat more fruit and veg      stop or cut down drug use      Find ways to feel less sad or depressed      None of these(76  Question Name = When change Weight)When do you think you will try to lose weight?      I'm already trying to lose weight      In the next month      In the next 2-6 months      Sometime, but not in the next 6 months(77  Question Name = When change Smoking)When do you think you will try to stop or cut down smoking?      I'm already trying to stop/cut down smoking      In the next month      In the next 2-6 months      Sometime, but not in the next 6 months(78  Question Name = When change Alcohol)When do you think you will start to try to drink less alcohol?      I'm already trying to drink less alcohol      In the next month      In the next 2-6 months      Sometime, but not in the next 6 months(79  Question Name = When change Exercise)When do you think you will start to try to get more exercise?      I'm already trying to do more exercise      In the next month      In the next 2-6 months      Sometime, but not in the next 6 months(80  Question Name = When change Fruit and veg)When do you think you will start to try to eat more fruit and veg?      I'm already trying to eat more fruit and veg      In the next month      In the next 2-6 months      Sometime, but not in the next 6 months(81  Question Name = When change Drugs)When do you think you will try to stop or cut down drug use?      I'm already trying to stop or cut down on drug use      In the next month      In the next 2-6 months      Sometime, but not in the next 6 months(82  Question Name = When change Depression)When would you like help to change feelings of sadness or depression?      I'm already getting help      In the next month      In the next 2-6 months      Sometime, but not in the next 6 months(83  Question Name = Approach)If you could get help (eg from your doctor or a health worker), what would be the best way for you to make these changes?      I would finish making one change before I started on the next one      Once I started to get somewhere with one change, I would start on the next one      I would try to make some or all of these changes at once(84  Question Name = Approach Same)Which changes would you try to make at the same time?      Lose weight      Stop or cut down smoking      Drink less alcohol      Get more exercise      Eat more fruit and veg      Stop or cut down on drug use      Work on feelings of depression or sadness(85  Question Name = Assistance AMS Weight)Choose as many types of help as you would useWould you use any of these services to help you lose weight?      Advice and help from my doctor, who checks how I'm going      Advice and help from a Health Worker, who checks how I'm going      My doctor or Health Worker arranging for me to see a specialist (like a dietician or exercise coach)      I arrange to see a specialist myself (like a dietician or exercise coach)      None of these(86  Question Name = Self or More Weight1)It would be best if this help:      Was just for me      Was for me and ONE support person (like my partner, a parent or sibling, or a close friend)      Was for me and other members of my family or my friends      Not sure(87  Question Name = Assistance Other Weight)Choose as many types of help as you would useWould you use any of these services to help you lose weight?      Go to face-to-face support group meetings with others also trying to lose weight      Use a computer to get emails or on-line advice and support      Call a telephone service for advice and support      Take home books or DVDs with information and advice for losing weight      Use a phone app and text messages for advice and support      None of these(88  Question Name = Self or More Weight2)It would be best if this help:      Was just for me      Was for me and ONE support person (like my partner, a parent or sibling, or a close friend)      Was for me and other members of my family or my friends      Not sure(89  Question Name = Assistance AMS Smoking)Choose as many types of help as you would useWould you use any of these services to help you stop smoking?      Advice and help from my doctor, who checks how I'm going      Advice and help from a Health Worker, who checks how I'm going      My doctor or Health Worker arranging for me to see a specialist (like an expert in quitting or a counsellor)      I arrange to see a specialist myself (like an expert in quitting or a counsellor)      None of these(90  Question Name = Self or More Smoke1)It would be best if this help:      Was just for me      Was for me and ONE support person (like my partner, a parent or sibling, or a close friend)      Was for me and other members of my family or my friends      Not sure(91  Question Name = Assistance Other Smoking)Choose as many types of help as you would useWould you use any of these services to help you stop smoking?      Go to face-to-face support group meetings with others who are also trying to quit      Use a computer to get emails or on-line advice and support      Call a telephone service for advice and support      Take home books or DVDs with information and advice for quitting      Use a phone app and text messages for advice and support      None of these(92  Question Name = Self or More Smoke2)It would be best if this help:      Was given just to me      Was for me and ONE support person (like my partner, a parent or sibling, or a close friend)      Was for me and other members of my family or my friends      Not sure(93  Question Name = Assistance AMS Alcohol)Choose as many types of help as you would useWould you use any of these services to help you drink less alcohol?      Advice and help from my doctor, who checks how I'm going      Advice and help from a Health Worker, who checks how I'm going      My doctor or Health Worker arranging for me to see a specialist (like a drug and alcohol worker)      I arrange to see a specialist myself (like a drug and alcohol worker)      None of these(94  Question Name = Self or More Alcohol1)It would be best if this help:      Was just for me      Was for me and ONE support person (like my partner, a parent or sibling, or a close friend)      Was for me and other members of my family or my friends      Not sure(95  Question Name = Assistance Other Alcohol)Choose as many types of help as you would useWould you use any of these services to help you drink less alcohol?      Go to face-to-face support group meetings with others also trying to drink less alcohol      Use a computer to get emails or on-line advice and support      Call a telephone service for advice and support      Take home books or DVDs with information and advice for reducing alcohol      Use a phone app and text messages for advice and support      None of these(96  Question Name = Self or More Alcohol2)It would be best if this help:      Was just for me      Was for me and ONE support person (like my partner, a parent or sibling, or a close friend)      Was for me and other members of my family or my friends      Not sure(97  Question Name = Assistance AMS Exercise)Choose as many types of help as you would useWould you use any of these services to help you get more exercise?      Advice and help from my doctor, who checks how I'm going      Advice and help from a Health Worker, who checks how I'm going      My doctor or Health Worker arranging for me to see a specialist (like an exercise coach or trainer)      I arrange to see a specialist myself (like an exercise coach or trainer)      None of these(98  Question Name = Self or More Exercise1)It would be best if this help:      Was just for me      Was for me and ONE support person (like my partner, a parent or sibling, or a close friend)      Was for me and other members of my family or my friends      Not sure(99  Question Name = Assistance Other Exercise)Choose as many types of help as you would useWould you use any of these services to help you get more exercise?      Go to face-to-face support group meetings with others also trying to get more exercise      Use a computer to get emails or on-line advice and support      Call a telephone service for advice and support      Take home books or DVDs with information and advice for getting more exercise      Use a phone app and text messages for advice and support      None of these(100  Question Name = Self or More Exercise2)It would be best if this help:      Was just for me      Was for me and ONE support person (like my partner, a parent or sibling, or a close friend)      Was for me and other members of my family or my friends      Not sure(101  Question Name = Assistance AMS Fruit and veg)Choose as many types of help as you would useWould you use any of these services to help you eat more fruit and veg?      Advice and help from my doctor, who checks how I'm going      Advice and help from a Health Worker, who checks how I'm going      My doctor or Health Worker arranging for me to see a specialist (like a dietician)      I arrange to see a specialist myself (like a dietician)      None of these(102  Question Name = Self or More Fruit1)It would be best if this help:      Was just for me      Was for me and ONE support person (like my partner, a parent or sibling, or a close friend)      Was for me and other members of my family or my friends      Not sure(103  Question Name = Assistance Other Fruit and veg)Choose as many types of help as you would useWould you use any of these services to help you eat more fruit and veg?      Go to face-to-face support group meetings with others also trying to change their diet      Use a computer to get emails or on-line advice and support      Call a telephone service for advice and support      Take home books or DVDs with information and advice      Use a phone app and text messages for advice and support      None of these(104  Question Name = Self or More Fruit2)It would be best if this help:      Was just for me      Was for me and ONE support person (like my partner, a parent or sibling, or a close friend)      Was for me and other members of my family or my friends      Not sure(105  Question Name = Assistance AMS Drugs)Choose as many types of help as you would useWould you use any of these services to help you cut down or stop using drugs?      Advice and help from my doctor, who checks how I'm going      Advice and help from a Health Worker, who checks how I'm going      My doctor or Health Worker arranging for me to see a specialist (like a Drug and Alcohol worker)      I arrange to see a specialist myself (like a Drug and Alcohol worker)      None of these(106  Question Name = Self or More Drug1)It would be best if this help:      Was just for me      Was for me and ONE support person (like my partner, a parent or sibling, or a close friend)      Was for me and other members of my family or my friends      Not sure(107  Question Name = Assistance Other Drugs)Choose as many types of help as you would useWould you use any of these services to help you cut down or stop using drugs?      Go to face-to-face support group meetings with others also trying to stop using drugs      Use a computer to get emails or on-line advice and support      Call a telephone service for advice and support      Take home books or DVDs with information and advice      Use a phone app and text messages for advice and support      None of these(108  Question Name = Self or More Drug2)It would be best if this help:      Was just for me      Was for me and ONE support person (like my partner, a parent or sibling, or a close friend)      Was for me and other members of my family or my friends      Not sure(109  Question Name = Depression Assistance AMS)Choose as many types of help as you would useWould you use any of these services to help with feeling depressed, sad or down?      Advice and help from my doctor, who checks how I'm going      Advice and help from a Health Worker, who checks how I'm going      My doctor or Health Worker arranging for me to see a specialist (like a psychologist)      I arrange to see a specialist myself (like a dietician or exercise coach)      None of these(110  Question Name = Self or More Depression1)It would be best if this help:      Was just for me      Was for me and ONE support person (like my partner, a parent or sibling, or a close friend)      Was for me and other members of my family or my friends      Not sure(111  Question Name = Depression Assistance other)Choose as many types of help as you would useWould you use any of these services to help with feeling depressed, sad or down?      Go to face-to-face support group meetings      Use a computer to get emails or on-line advice and support      Call a telephone service for advice and support      Take home books or DVDs with information and advice      Use a phone app and text messages frh advice and support      None of these(112  Question Name = Self or More depression2)It would be best if this help:      Was just for me      Was for me and ONE support person (like my partner, a parent or sibling, or a close friend)      Was for me and other members of my family or my friends      Not sure(113  Question Name = One thing)If you could choose ONE thing that would improve your life the most, what would you choose?      Being healthier      My family being healthier      Having better relationships with my family or friends      Better housing      Having a job or a better job      A better education or being able to study      Stop violence at home      Stop violence in my community      Stop crime in my community      Stop racism or discrimination      Not sure      None of these(114  Question Name = Finish screen)
