# Supplementary material for: A randomised trial assessing the acceptability and effectiveness of providing generic versus tailored feedback about health risks for a high need primary care sample
Source: BMC Fam Pract. 2015 Aug 5;16:95. doi: 10.1186/s12875-015-0309-7 (PMC4525725; doi:10.1186/s12875-015-0309-7)
Supplement: Additional file 4: — Example of the generic and tailored feedback. [file 12875_2015_309_MOESM4_ESM.rtf]

English text of the survey: Durri ACMS Exit Survey

(1  Question Name = Info Screen)


(2  Question Name = Participant ID)
Enter participant ID:

(3  Question Name = Talk about topics)
Touch all that apply, then touch NEXT
Did you and your doctor or health worker TALK about any of these health topics in your appointment today?
      Weight
      Smoking
      Alcohol
      Diet
      Exercise
      Drugs
      None of these topics
      I prefer not to say

(4  Question Name = Talk about topics2)
Touch all that apply, then touch next
Did you and your doctor or health worker TALK about any of these health topics in your appointment today?
      Depression
      BP
      Cholesterol
      Diabetes
      Cancer
      None of these topics
      I prefer not to say

(5  Question Name = Did checklist help)
Touch all that apply, then touch NEXT
Did the checklist we gave you help you to talk about any of those topics?
      Yes- I gave the checklist to the doctor
      Yes- the checklist gave me some ideas about what to ask the doctor
      No- I didn't use the checklist
      Not sure

(6  Question Name = Action)
Touch all that apply, then touch NEXT
Thinking about the health topics from the survey (pictured), did your doctor do any of these things today?
      Gave me information (e.g. website or pamphlets)
      Helped me plan changes to my lifestyle
      Organised a follow up appointment for me
      None of these things

(7  Question Name = Action2)
Touch all that apply, then touch NEXT
Thinking about the health topics on the checklist (pictured), did your doctor or health worker do any of these things today?
      Gave me a test
      Booked or gave me a referral for a test
      Prescribed a medication for me
      Something else
      My doctor didn't do anything else

(8  Question Name = Checklist feedback)
This question is about the checklist that we gave you.

I think that the checklist:
      Was easy to understand
      Was relevant to me
      Will help me improve my health
      Yes
      No
      Not sure

(9  Question Name = Feedback preferences)
Please choose a number between 1 (very good) and 5 (very bad)
Overall, do you think that doing this survey, and getting a checklist, is a good way to help people to improve their health?
      
      A very GOOD way
      
      Not sure
      
      A very BAD way

(10  Question Name = Finish screen)
